# Announcing the 2018 *Medicines* Travel Award for PostDocs

**DOI:** 10.3390/medicines5010009

**Published:** 2018-01-16

**Authors:** Gerhard Litscher

**Affiliations:** Editor-in-Chief of *Medicines*, Head of the Research Unit for Complementary and Integrative Laser Medicine, of the Research Unit of Biomedical Engineering in Anesthesia and Intensive Care Medicine, and of the TCM Research Center Graz, Medical University of Graz, 8036 Graz, Austria; gerhard.litscher@medunigraz.at; Tel.: +43-316-385-13907

For the *Medicines* Travel Award 2018, we received a total of 41 applications from all over the world, of a very high quality. After preselection from the managing editorial staff of *Medicines*, scientific decision has been performed by four experts from Asia, Australia, and America and by the editor-in-chief of *Medicines* from Europe. Therefore, the jury included experts from four different continents.

As Editor-in-Chief of *Medicines*, I am pleased to announce the winner of the *Medicines* Travel Award for 2018. The travel award was granted to Dr. Amie Steel, a postdoctoral research fellow at the Australian Research Center in Complementary and Integrative Medicine at the University of Technology in Sydney, Australia. The award consists of 800 Swiss Francs to attend any academic conference during 2018.


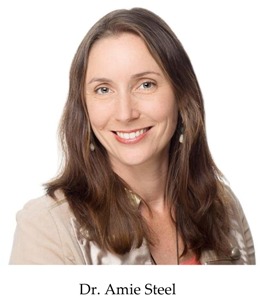


Dr. Steel is also Associate Director Research at Endeavour College of Natural Health. In the four years since she has received her PhD, she has published over 100 research contributions, including 75 journal articles, two books, and 20 book chapters. She has attracted a large amount of research funding for complementary medicine (CM) research in the last 12 months, the majority of which is directed towards clinical trials involving medicinal cannabis and other natural health products.

Her research interests fit within the broad remit of health services research, with a particular focus on understanding clinical practice and supporting clinical research which is embedded in and reflects daily routine care. She is a member of the Steering Committee for three national CM practice-based research networks in Australia, and she has led the formation of the International Research Collaborative for Naturopathic Academic Clinics—a network of research sites in naturopathic educational institutions which crosses four countries. This research infrastructure supports pragmatic clinical research within CM. She is also a peer mentor on the ARCCIM Research Leadership Programs for both CM and Naturopathy, and is a member of the World Naturopathic Federation Research Committee through which she has also contributed to policy development for the World Health Organization.

In recent years, she has turned her attention to the evolving field of implementation science. In particular, she is interested in exploring the most effective way to integrate and implement different forms of knowledge. Within CM scholarship, the concept of the “embodied knowledge of the experienced clinician” is defended and criticized in equal measure, and she would like to explore the possibility of drawing upon the strategies being developed within implementation science to address this challenge within her own topic area.

Congratulations to Dr. Amie Steel on behalf of the international jury and the *Medicines* editorial and publishing teams! 

